# Supplementary material for: Mapping climate change’s impact on cholera infection risk in Bangladesh
Source: PLOS Glob Public Health. 2022 Oct 14;2(10):e0000711. doi: 10.1371/journal.pgph.0000711 (PMC10021506; doi:10.1371/journal.pgph.0000711)
Supplement: S1 Text — (DOCX) [file pgph.0000711.s001.docx]

**Supplementary Materials**

**Mapping Climate Change's Impact on Cholera Infection Risk in Bangladesh**

Sophia E. Kruger, Paul A. Lorah, Kenichi W. Okamoto

**S1. Spatial Data Manipulation in R and ArcGIS**

To interface R programming language with ArcGIS Pro, a Geographic Information Systems (GIS) software, we used the ‘arcgisbinding’ package in R to facilitate loading ArcGIS raster layers into R and exporting raster layers from R to ArcGIS [1]. All raster data available from source as TIFF files were uploaded into ArcGIS Pro, projected to WGS84, resampled to a resolution of 250m square grid cells, and cropped to the rectangular extent (88.01057°W, 92.67366°E, 20.74111°S, 26.63407°N) surrounding the country of Bangladesh. Image service layers, provided by ESRI’s Living Atlas Portal, were imported into ArcGIS through the portal, then projected, resampled, and cropped with the same procedure as described for all TIFF raster files.

Of the covariates used for 2015, the average precipitation and temperature raster layers were products of additional data manipulation that occurred in R and ArcGIS Pro. For 2050, the precipitation, temperature, and elevation rasters required additional manipulation. Below we detail additional steps taken to include these data as spatial covariates in our model.

*S1.1 Average Precipitation and Temperature Rasters (2015 and 2050)*

The average precipitation and maximum and minimum temperature rasters were created from average monthly climate data (as NetCDF files) provided by TerraClimate [2]. The NetCDF files were converted in R to GeoTIFF files (see “file-coversion.R” in repository), then exported to ArcGIS for projecting, resampling, and cropping using the ModelBuilder functionality. Using the raster calculator tool in ModelBuilder, the raster bands for October 2015 through January 2016 were averaged to estimate the average monthly precipitation accumulation (mm), maximum temperature (°C), and minimum temperature (°C) experienced in Bangladesh during the survey period of the Azman et al. (2020) data [3]. The months were selected with the assumption that the climatic conditions during the survey period would have influenced measured incidence. To consider the possibility that cholera incidence is a lagging indicator predicted by rainfall and temperatures of the monsoon season, we also created separate monsoon temperature and precipitation covariates for 2015 using the same methodology to average the monthly values for June through September. This process of averaging the precipitation and temperature data in ArcGIS was also replicated in R (see “2015-raster-manipulation.R” in repository) to ensure that estimated averages were consistent across platforms. For 2050, the average precipitation and maximum and minimum temperature rasters from WorldClim (v2.1) were created following the same procedure as described for the TerraClimate files, though these layers were downloaded from source as TIFF files and thus did not need conversion (see “2050-raster-manipulation.R” for the averaging process in R) [4].

*S1.2 Elevation Raster (2050)*

The 90-meter coastal elevation layer from Kulp and Strauss (2018) at ClimateCentral was projected, resampled, and cropped to Bangladesh in ArcGIS [5]. The raster layer was then exported to R wherein missing values were imputed from the 2015 elevation layer using a generalized linear model in R (see “2050-raster-manipulation.R” in repository).

Once all layers were manipulated using the ModelBuilder functionality in ArcGIS, all raster layers were exported for use in R. To avoid memory overload issues in using R solely to project, resample, and crop global raster layers, our methodology relies on ArcGIS’s ModelBuilder to do the same and export all raster layers to R for analysis. Nonetheless, the code written in R follows the procedure streamlined in ArcGIS (e.g., projecting, resampling, and cropping) to encourage reproducibility and to also ensure that all raster layers, once imported into R, align to the desired raster cell size, global projection, and study area extent (see “2015-raster-manipulation.R” and “2050-raster-manipulation.R” in repository).

**References**

1. ESRI (2019). arcgisbinding: Bindings for ArcGIS. R package version 1.0.1.237. http://esri.com/.

2. Abatzoglou, John T., Solomon Z. Dobrowski, Sean A. Parks, and Katherine C. Hegewisch. "TerraClimate, a high-resolution global dataset of monthly climate and climatic water balance from 1958–2015." *Scientific Data* 5, no. 1 (2018): 1-12.

3. Azman, A. S., Lauer, S. A., Bhuiyan, T. R., Luquero, F. J., Leung, D. T., Hegde, S. T., ... & Gurley, E. S. (2020). *Vibrio cholerae* O1 transmission in Bangladesh: insights from a nationally representative serosurvey. *The Lancet Microbe*, 1(8), e336-e343.

4. CMIP6 Downscaled Monthly Climate Projections: 2041-2060. WorldClim v2.1.

5. Kulp, Scott A., and Benjamin H. Strauss. "CoastalDEM: a global coastal digital elevation model improved from SRTM using a neural network." *Remote Sensing of Environment* 206 (2018): 231-239.

Supporting Information Legends

Figure S1. Response curve of the 2015 occurrence probabilities generated by our bivariate random forest model plotted against the elevation values for 2015.

Figure S2. Response curve of the 2015 occurrence probabilities generated by our bivariate random forest model plotted against the distance to water values for 2015.

Figure S3. Mean and quantile (2.5% and 97.5%) risk maps predicting cholera infection risk for 2015 (a-c) and 2050 (d-e) from predictions constructed by our best-fitting random forest model. Risk values range from 0 to 1 with 1 representing the highest risk for cholera infection in the specified geographic area. Base map: [World Topographic Map](https://www.arcgis.com/home/item.html?id=7dc6cea0b1764a1f9af2e679f642f0f5). Base map credits: Esri, HERE, Garmin, FAO, NOAA, USGS, © OpenStreetMap contributors, and the GIS User Community.

Table S1. Confidence interval (95%) of the variable importance (mean decrease in Gini) for each covariate included in our full model. A higher mean decrease in Gini coefficient reflects a variable’s greater importance to the random forest model. The (M) differentiates our temperature and precipitation variables between data taken during the monsoon period (M) and not.

Table S2. Change in AUC confidence interval for each model produced in the stepwise model selection process.
